# Effects of Different Numbers of Trials on Saccadometry Test Results

**DOI:** 10.1002/brb3.70700

**Published:** 2025-07-26

**Authors:** Aysenur Kucuk Ceyhan, Asya Fatma Men, Zahra Polat

**Affiliations:** ^1^ Department of Audiology, Faculty of Health Sciences University of Health Sciences Istanbul Turkey

**Keywords:** antisaccades, number of trials, prosaccades, saccadometry test protocols, saccadometry

## Abstract

**Purpose:**

The aim of this study was to investigate the effect of the number of trials on the recording in the saccadometry test.

**Method:**

Forty‐five healthy participants (mean age ± SD = 25.89 ± 5.2 years) (31 female and 14 male) aged 19–40 years were included in the study. Each participant underwent two saccadometry tests, first a test protocol with 100 trials and then a test protocol with 60 trials, administered by the same experienced clinician, one hour apart. All test settings remained constant between the two test sessions, with the exception of the number of trials.

**Results:**

With 100 trials, antisaccades had a much higher mean latency (paired samples *t*‐test; *t* = 4.838; *p* = 0.0001 < 0.01), directional error rate (Wilcoxon signed ranks test; *Z* = −1.991; *p* = 0.047), and overall error rate (Wilcoxon signed rank test; *Z* = −2.207; *p* = 0.027) compared to the results obtained from the test protocol with 60 trials. There was no significant difference in mean velocity or accuracy (Wilcoxon signed rank test; *p* > 0.05). The prosaccades, mean latency, velocity, directional error, and overall error (Wilcoxon signed ranks test; *p* > 0.05) and mean accuracy (paired samples *t*‐test; *p* > 0.05) did not differ between 100 and 60 trials.

**Conclusion:**

The decline in antisaccade performance with an increasing number of trials may be attributed to the disruptive effect of mental fatigue on the inhibition process. Further research is required to investigate the relationship between mental fatigue and the inhibition process in the context of antisaccade function.

## Graphical abstract

This study found that increasing the number of trials in the saccadometry test significantly impaired antisaccade performance by increasing latency and error rates, while prosaccade results remained stable. The findings highlight the importance of optimizing trial numbers in antisaccade testing for accurate assessment of eye movement control.

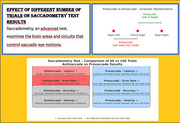

## 1 Introduction

In cases of abnormal functioning of core executive functions, eye movements can be altered in various ways. The ability to document and analyze eye movements serves as a crucial tool for understanding the functional integrity of brain systems (Hutton and Ettinger 2006). Saccades, which are rapid eye movements, are essential for shifting the visual system's focus abruptly to an object (Erkelens 2006). Saccadometry is an advanced eye movement analysis that assesses the brain regions and circuits responsible for generating precise saccadic movements (Munoz and Everling 2004).

Saccadometry includes two types of saccade tests: prosaccades and antisaccades. A prosaccade is a movement directed toward a presented target, whereas an antisaccade is a movement produced toward the opposite side of a presented target (Hallet 1978). Prosaccade is an automatic eye movement toward a visual target. Performing an antisaccade requires the inhibition of the automatic eye movement toward the target and the planning of a deliberate saccade in the opposite direction. (Ting et al. 2016; Munoz et al. 2003). Inhibitory control is defined as the ability to suppress unwanted thoughts, emotions, and behaviors (Diamond 2013).

Antisaccade performance engages a broader neural network than prosaccades, integrating executive control, motor planning, sensory processing, and inhibitory control centers. In contrast, prosaccades are primarily governed by motor control mechanisms and require minimal inhibitory processing, as they are largely automatic and stimulus‐driven (Munoz and Everling 2004). During antisaccade generation, neural networks involving cortical and subcortical regions become active, including the frontal eye field (FEF) for voluntary saccade control, the superior colliculus (SC) for integrating motor commands, the dorsolateral prefrontal cortex (DLPFC) for inhibitory control, the lateral intraparietal area (LIP) for spatial processing, the anterior cingulate cortex (ACC) for conflict detection, and the supplementary eye field (SEF) and pre‐supplementary motor area (pre‐SMA) for rule‐based motor execution. FEF is a region involved in the voluntary control of eye movements. Saccade‐related neurons within the FEF send direct projections to SC and can initiate saccadic commands. SC initiates saccadic eye movements by integrating incoming cortical signals and sensory inputs. In the antisaccade task, the activity of SC neurons on the stimulus side is suppressed, while the region of the SC on the opposite side of the stimulus is activated to execute the voluntary antisaccadic movement. DLPFC plays a central role in functions such as working memory, rule‐following, and behavioral inhibition. During an antisaccade, DLPFC is crucial for suppressing the tendency to look at the stimulus and enforcing the “look in the opposite direction” rule. The DLPFC sends direct projections to the SC. The basal ganglia, particularly the substantia nigra pars reticulata and the internal segment of the globus pallidus, exhibit tonic inhibition and suppress motor structures such as the SC to prevent unwanted reflexive saccades and facilitate the execution of the desired antisaccade. LIP is crucial for visual attention and the representation of target location. During an antisaccade, the LIP encodes the stimulus location while also contributing to the planning of the intended saccade to the mirrored position. ACC is associated with functions such as error monitoring, conflict detection, and motivational persistence. In the antisaccade task, ACC is thought to detect the conflict between prosaccade and antisaccade commands and contribute to the selection of the correct response. SEF and the adjacent pre‐SMA are particularly involved in rule‐based and sequential movement control. Increased SEF activity has been observed during rule‐based tasks such as the antisaccade task. Given that the pre‐SMA/SEF region is generally involved in voluntary movement initiation and rule implementation, its activation during the antisaccade task supports the subject's execution of the internal rule (looking in the opposite direction) (Kimmig et al. 2001, Munoz and Everling 2004).

Mental fatigue is a subjective feeling of exhaustion or a perceived decline in cognitive ability that individuals experience during or after prolonged cognitive activity requiring sustained mental efficiency (Lorist et al. 2005). It has been shown that the structures responsible for controlling executive functions involved in the antisaccade mechanism exhibit a decline in function due to mental fatigue (Ferreira et al. 2024; Wójcik and Maj 2025; Ahn et al. 2023; Halliday and Carpenter 2010). In light of these findings, we hypothesize that increasing the number of trials and consequently extending the test duration may have an impact on the results of the antisaccade test. The antisaccade test can be administered with different trial numbers, where “trial number” refers to the total count of individual attempts a participant makes in the task. Antoniades et al. (2013) highlighted two opposing perspectives regarding the number of trials. Some researchers argue that tests increasing error rates are necessary to highlight pathological differences. However, Antoniades et al. (2013) suggested that overly demanding tasks may generate excessive errors, reducing their diagnostic value, while overly simple tasks may produce too few errors to allow for an effective assessment. Since the effectiveness of the saccadometry test has been demonstrated in the early diagnosis and monitoring of neurological disorders such as Alzheimer's disease and mild cognitive impairment (Opwonya et al. 2022) and Parkinson's disease (Waldthaler et al. 2021), the number of trials used in saccadometry assessments emerges as a crucial parameter. However, to the best of the authors’ knowledge, no study has investigated the impact of varying trial numbers on saccadometry outcomes. This study aims to evaluate the effects of varying trial numbers and extended test durations on saccadometry results in young and healthy individuals. Examining the effects of variations in trial numbers in young and healthy individuals may provide preliminary insights for future studies involving pathological groups.

## 2 Materials and Methods

### 2.1 Participants

The sample included 45 (min–max = 19–40, mean age ± SD = 25.89 ± 5.2 years), 31female (68.9%) and14 male (31.1%) healthy participants. Data collection was carried out at the University of Health Sciences Audiology Laboratory in Istanbul between December 2023 and January 2024. Participants were selected from among the students studying at the University of Health Sciences and the staff of the hospital via the voluntary sampling method. The sample size was determined for at least 90% power with a medium effect size of 0.5 and a margin of error of 0.05 using the GPower 3.1 program as minimum 40 individuals. Participants with a history of balance, visual, neurological, otological, psychiatric, cognitive, or systemic impairments; hearing loss; or the use of centrally acting medications were excluded to avoid potential interference with saccadic measurements. All included participants demonstrated intact central vestibular function based on oculomotor testing and achieved a minimum score of 24 on the Mini‐Mental State Examination. This study was approved by the Medical Research Ethics Committee of the University of Health Sciences (date: May 18, 2023, decision No.: 10/11). Written informed consent was given by all participants.

### 2.2 Procedures

Pure tone audiometry and oculomotor tests were conducted to determine compliance with the inclusion criteria. Hearing loss is known to be a disruptive factor for executive functions due to the increased listening effort it causes (Meister et al. 2016). Although its exact impact on antisaccade measurements is not fully understood, individuals with hearing loss were excluded from the study due to its potential confounding effects. Audiometric tests were performed in quiet rooms at IAC (Industrial Acoustic Company) standards using a clinical audiometer (Madsen Astera; Denmark). Air conduction hearing thresholds were measured in the range of 125–8000 Hz. Oculomotor tests were performed using videonystagmography (VNG) (Interacoustics VisualEyes 525; Denmark). VNG is a vestibular assessment of peripheral vestibular systems located in the inner ear and the central motor functions of eye movement. Spontaneous nystagmus, gaze, smooth pursuit, random saccades, optokinetic nystagmus tests found within the VNG test battery were performed to exclude central vestibular system disfunctions.

### 2.3 Saccadometry

#### 2.3.1 Basic Test Administration

Saccadometry tests were conducted using a VNG system (Interacoustics VisualEyes 525). Participants were seated in a stable chair positioned 1 m from a screen. The screen's background was black, with red stimuli targets occupying 1% of the screen's total width. Each stimulus appeared at a consistent distance of 10° to the left or right of the fixed central target, following a random delay of 1–2 s (mean interval: 1.5 s). Tests were conducted in the horizontal plane. In the study, the number of dots was set to two. When the number of dots is set to two, the center target is displayed first, followed by a brief appearance of the target position. The patient first looks at the center target, then looks at the flashing target for the prosaccade task or in the opposite direction of the flashing target for the antisaccade task, and finally returns to the center position. The center target is throughout the test. Since the fixation point is not removed before the new target appears, the gap paradigm does not occur. Under this condition, the occurrence of anticipatory saccades is reduced. VNG calibration was performed at the beginning of the test battery. Participants wore VNG goggles to record eye movements, with adjustments made to cameras, mirrors, and focus to optimize pupil detection and minimize artifacts. To reduce response‐related artifacts, participants were instructed to keep their eyes open, maintain head stability, and adhere to given instructions. Artifact rejection was enabled for all tests.

#### 2.3.2 Prosaccade and Antisaccade Test Parameters

In the prosaccade test, participants were instructed to look directly at the illuminated dot on the screen as it appeared. In the antisaccade test, participants were instructed to look in the direction opposite to the illuminated dot on the screen, aiming their gaze at an approximately symmetrical point relative to the target. The first test was conducted as a block trial, consisting of 100 trials (50 right, 50 left) with a total duration of 256 ms. In a block trial, the same test parameters (either only prosaccade or only antisaccade) are used throughout the entire trial (Antoniades et al. 2007). In the saccadometry test, switching between tasks is associated with a reconfiguration cost (Aponte et al. 2019). In this study, task difficulty was linked to an increase in the number of trials and the extension of the recording duration. Accordingly, the prosaccade test was completed first, consisting of 50 right and 50 left trials, followed by the antisaccade test with 50 right and 50 left trials for all participants. We gave a 3‐ to 4‐min break between the two tests, during which we asked the participants if they were ready. The antisaccade test was then initiated, with the necessary instructions provided. After a one‐hour rest period, a second test was administered following the same protocol but with 60 trials (30 right, 30 left) and 151 ms total duration. Both tests were conducted on the same day to minimize the influence of person‐dependent factors such as illness or sleep deprivation. A one‐hour interval was maintained between the 100‐trial and 60‐trial tests. During the rest period, participants relax in the waiting room or the garden. Before starting the second test, participants were asked again if they were ready to proceed, and all responded positively before being taken to the test room.

At the conclusion of each test, the following parameters were evaluated: *Latency* (*ms*): Measures the time between the visual stimulus and the initiation of the saccadic movement. *Peak velocity* (*degrees per second*): Indicates the velocity at which the eyes move from one point to another. *Accuracy* (*percentage*): Assesses the ability to move the eyes directly toward the target without overshooting or undershooting. Impairments in inhibitory mechanisms, along with dysfunctions in the basal ganglia, parietal cortex, and specific cerebellar systems, can lead to inaccurate target identification and deviations in eye movements. *Directional error rate*: The percentage of instances where the eyes moved in the incorrect direction. *Overall error rate*: Reflects the overall noise and artifacts present in the recording (Munoz and Everling 2004).

The device used in the study automatically calculates outcome measures within the defined framework. Saccade latency is calculated as the time from the target movement until the threshold velocity is reached. The saccade ends when the velocity drops below the threshold again. Peak velocity is identified within the period between the start and end of the saccade. Accuracy is determined as the ratio between the amplitude of the target movement and the amplitude of the eye movement. The amplitude of the eye movement is calculated as the difference between the eye position before and after the saccade. The pre‐saccade position is determined as the average position during the latency period. The post‐saccade position is determined as the average position during the Accuracy Search Period.

**FIGURE 1:**
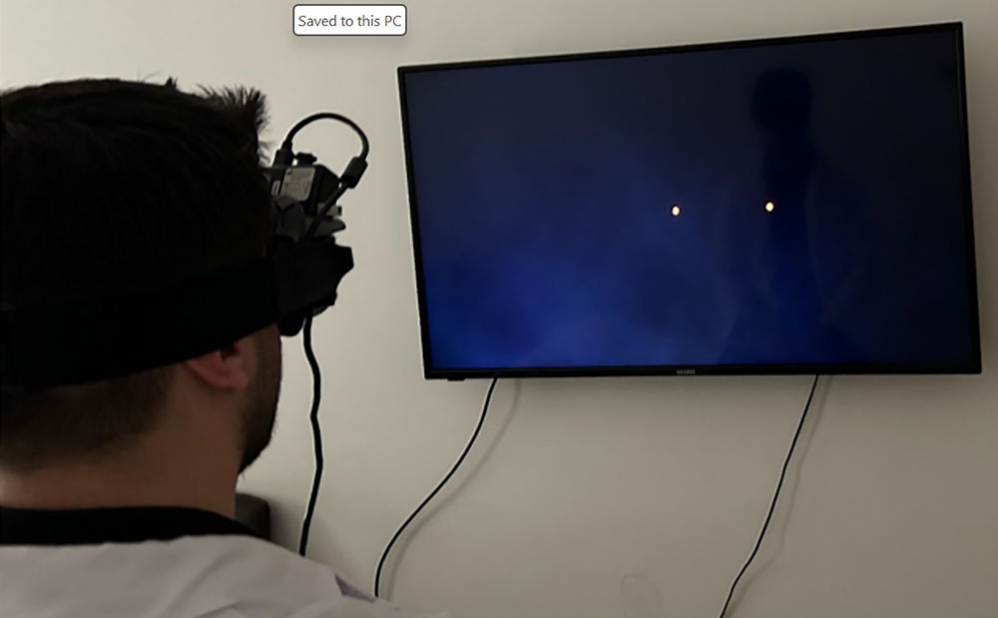
*Experimental setup*: On a black screen positioned 1 m in front of the patient, a red center dot appears first, followed by a second dot that appears either to the left or right of the center dot. During the prosaccade test, the patient should look toward the second dot. During the antisaccade test, the patient should look in the opposite direction of the second dot. The central dot remains illuminated throughout the test, while the second dot appears following a random delay of 1–2 s (mean interval: 1.5 s).

**FIGURE 2:**
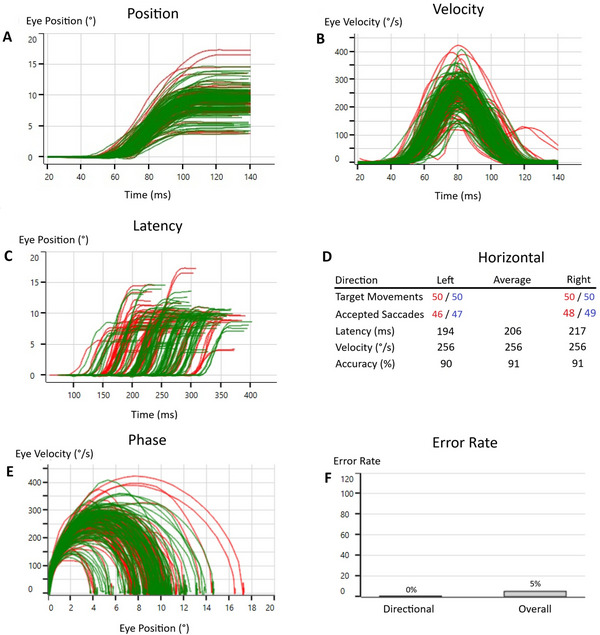
*Test analysis screen*: Graphical representations of individual eye and target direction data enable assessment of potential disconjugate eye movements. Leftward saccades are shown in red and rightward saccades in green. (A) Position: Indicates whether the eyes accurately reach the target and how long it takes to do so. (B) Velocity: Shows how fast the eyes move over time while approaching the target. (C) Latency: Reflects the time taken to initiate eye movement toward the target. (D) Numerical data: Provides quantitative measures including the number of target movements, accepted saccades, mean latency, peak velocity, and accuracy for leftward, rightward, and average directions. (E) Phase: Demonstrates the speed of eye movements relative to their position along the trajectory. (F) Error rate: Displays both directional and overall error rates. The directional error rate refers to the number of incorrect gaze directions, while the overall error rate includes both directional errors and artifacts, offering insight into signal quality.

**FIGURE 3:**
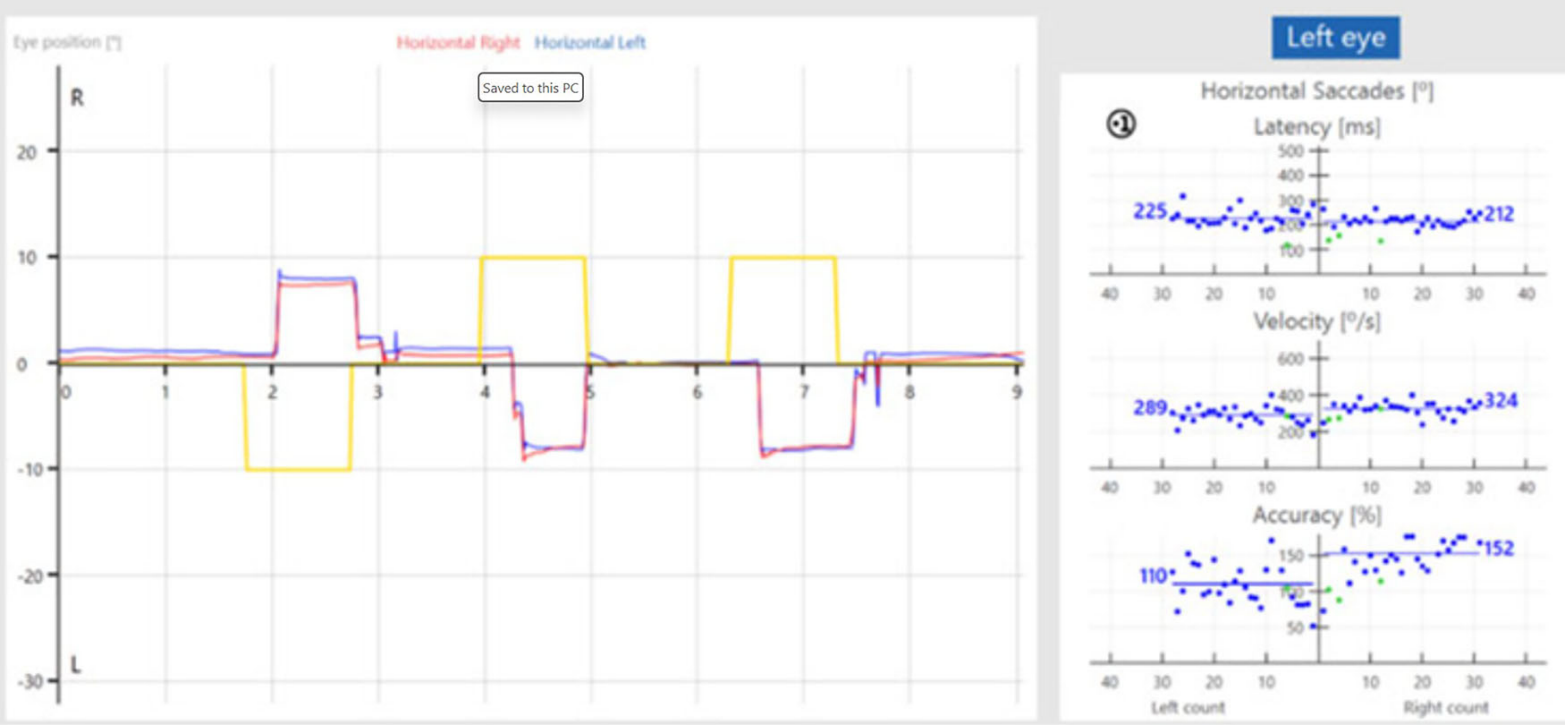
*Antisaccade data collection screen*: Eye movement data for the antisaccade test show that the eyes move in the opposite direction of the stimulus. The yellow line indicates the stimulus direction, while the red and blue lines represent the right and left eye, respectively. The image used in the figure is obtained from https://www.interacoustics.com.

The saccadometry test provided separate and average values for the right and left eyes across these parameters. For this study, average values encompassing both eyes and both target directions were subjected to statistical analysis. The test setup for the saccadometry test is presented in Figure 1, the analysis screen is presented in Figure 2, and the antisaccade data collection screen is presented in Figure 3.

### 2.4 Statistical Analysis

The IBM SPSS 26.0 package program was used for statistical analysis in this study. Descriptive statistics (frequency, percentage, median, interquartile range, minimum‐maximum values, mean, and standard deviation) were calculated for the demographic data of the participants and the data from the two different protocols. The distribution of the variables was determined with the Shapiro–Wilk test. The paired samples *t*‐test was used for normally distributed variables and the Wilcoxon signed ranks test for non‐normally distributed variables to compare mean latency, velocity, accuracy, directional error rate (%), and overall error rate (%) in prosaccades and antisaccades. All statistical analyses were performed at a 95% confidence interval, and significance was evaluated at *p* < 0.05.

## 3 Results

Table 1 presents a comparison of the results from the two different test procedures.

**TABLE 1:** The comparison of two different test procedures (100 trials and 60 trials).

		100 trials			60 trials			*p*
		Median (IQR)	Min–Max	Mean ± SD	Median (IQR)	Min–Max	Mean ± SD	
**Prosaccade (*n* = 45)**	**Mean latency (ms)**	243 (38)	186—349	243.28 ± 28.3	245 (47)	181–307	244.86 ± 28.6	0.495^b^
	**Mean velocity (^0^/s)**	280 (28)	179–426	277.05 ± 34.9	276 (32)	217–405	277.63 ± 31.3	0.87^b^
	**Mean accuracy (%)**	96 (7)	85–105	95.84 ± 4.9	96 (8)	82–106	96.44 ± 5.6	0.629^a^
	**Directional error rate (%)**	0 (1)	0–3	0.4 ± 0.66	0 (0)	0–4	0.33 ± 0.94	0.245^b^
	**Overall error rate (%)**	4 (4)	0–31	5.35 ± 5.97	3 (5)	0–32	5.12 ± 6.42	0.422^b^
**Antisaccade (*n* = 45)**	**Mean latency (ms)**	324 (46)	257—425	327.72 ± 35.4	305 (44)	201–378	303.3 ± 36.6	0.0001**^a^
	**Mean velocity (^0^/s)**	231 (59)	160—395	233.63 ± 48.6	231 (54)	169–451	238.91 ± 53.2	0.795^b^
	**Mean accuracy (%)**	84 (24)	58–132	86.84 ± 18.7	86 (20)	58–136	86.23 ± 14.6	0.836^b^
	**Directional error rate (%)**	7 (5)	0–27	7.56 ± 6.64	3 (7)	0–33	6.33 ± 7.44	0.047*^b^
	**Overall error rate (%)**	11 (8)	2–36	13.26 ± 8.38	8 (10)	0–73	12.07 ± 12.45	0.027*^b^

*Note*: There were no significant differences in the latency, velocity, directional error, overall error (Wilcoxon signed ranks test; *p* > 0.05), and accuracy (paired samples *t*‐test; *p* > 0.05) between 100 and 60 trials for prosaccades. Antisaccades had significantly higher latency (paired samples *t*‐test; *t* = 4.838; *p* = 0.0001), directional error rate (Wilcoxon signed ranks test; *Z* = −1.991; *p* = 0.047), and overall error rate (Wilcoxon signed ranks test; *Z* = −2.207; *p* = 0.027) when the protocol was set to 100 trials compared to 60 trials. There were no significant differences in velocity and accuracy between 100 and 60 trials (Wilcoxon signed ranks test; *p* > 0.05).

^a^ Paired samples *t* test.

^b^ Wilcoxon signed ranks test; *p < 0.05; **p < 0.01.

## 4 Discussion

In this study, the same young, healthy individuals underwent the saccadometry test twice using two different test protocols: one with 60 trials and the other with 100 trials. The results were compared in terms of latency, velocity, accuracy, overall error rate, and direction errors. Based on the assumption that an increase in the number of trials may induce mental fatigue (Milham et al. 2003; Lorist et al. 2005), this study investigated whether this increase would lead to impairments in measurement parameters in young and healthy individuals. While the prosaccade task in the saccadometry test did not show a significant difference between 60 and 100 trials, changes were observed in the antisaccade task results. As the number of trials increased in the antisaccade task, there was a significant increase in latency, direction errors, and overall errors, whereas no changes were found in velocity or accuracy.

### 4.1 Effects of Varying Trial Numbers on Antisaccade Task Performance

Differences in antisaccade test results were observed between 60 and 100 trials. With an increasing number of trials, latency, directional errors, and overall errors significantly increased, while velocity and accuracy remained unchanged. Consistent with our study, the literature indicates that in cases of functional weakness of the prefrontal cortex, error rates and latency are the parameters that show significant impairments (Munoz and Everling 2004). Longer antisaccade latencies and high error rates are thought to reflect inhibitor control efficiency (Derakshan et al. 2009).

Performing the antisaccade task calls for different kinds of brain activity, including activity in the prefrontal cortex (Rosano et al. 2002; Tu et al. 2006; Munoz and Everling 2004). The main research question of the study was whether mental fatigue, which is known to have a detrimental effect on executive functions involved in the antisaccade mechanism (Ferreira et al. 2024; Wójcik and Maj 2025; Ahn et al. 2023), is associated with the decrease in performance that occurs with an increase in the number of trials in the antisaccade test.

According to the procedure applied in our study, an increase in the number of trials led to a greater number of task repetitions and an extended task duration. As a result, participants had to sustain their attention and cognitive resources for a longer period. Berggren (2011) described the performance decline observed during the antisaccade task as “antisaccadic cost,” a concept measured by longer reaction times and higher error rates in antisaccade responses compared to prosaccade responses. Additionally, the same study reported that when an additional cognitive load was introduced, antisaccade reaction times significantly increased, whereas prosaccade reaction times remained unaffected. The researchers attributed this finding to the presence of an additional cognitive “cost” during the antisaccade task, arising from the engagement of inhibitory and other cognitive control processes. Demian et al. (2023) conducted a normalization study for the saccadometry test using the same test device used in our study. Their results indicated that, across all age groups, the antisaccade test produced more direction errors, longer latency, and lower velocity compared to the prosaccade test. Referring to Liu, they explained these differences by suggesting that the saccadic eye movement system is influenced by a wide range of cognitive factors, including attention, learning, working memory, and decision‐making processes. Indeed, it is well established that the percentage of correct responses in the antisaccade task is significantly reduced in conditions associated with frontal lobe dysfunction (Hellmuth et al. 2012).

To provide a discussion on how an increase in the number of trials and, consequently, the extension of test duration affects performance decline in the antisaccade test, we reviewed studies investigating the relationship between executive functions involved in antisaccade generation and mental fatigue.

The ACC is a central structure responsible for controlling executive functions involved in the antisaccade mechanism. It plays a critical role in managing attentional resources and regulating cognitive effort. By engaging in error monitoring and performance evaluation, the ACC contributes to executive control processes. During the antisaccade task, when erroneous prosaccadic responses occur, the ACC detects these errors and facilitates the correction of future responses (Agam et al. 2010). Lorist et al. (2005) and Milham et al. (2003) reported that neural activity in the ACC changes as a function of task duration. According to these authors, such alterations in ACC activity may represent a potential underlying mechanism of mental fatigue. They proposed that prolonged cognitive activity could lead to a reduction in mesencephalic dopaminergic projections to the ACC, resulting in impaired performance monitoring and insufficient performance adjustments. Based on this explanation, the performance decline observed in prolonged antisaccade tasks may be linked to reduced ACC functionality. However, to the best of the authors' knowledge, there is no existing evidence in the literature specifying when task duration or repetition in the antisaccade task should be directly associated with a decline in ACC function. Further studies are needed to establish a clearer understanding of the relationship between extended antisaccade task duration and ACC functionality.

Another key center controlling executive functions involved in the antisaccade mechanism is DLPFC, which plays an active role in regulating motor responses and directing attention to the appropriate target. The DLPFC has direct connections with SC and FEF, allowing it to exert higher order inhibitory control. Damage to the DLPFC disrupts this inhibitory function, making it more difficult to suppress prosaccadic responses and leading to erroneous saccades (Munoz and Everling 2004). Mental fatigue has been shown to have a disruptive effect on the DLPFC, impairing its regulatory functions and inhibitory control (Soutschek and Tobler 2020). Another significant finding is that the connectivity of the DLPFC with other brain regions also changes with fatigue. In fatigued individuals, alterations in the connectivity of the DLPFC with the insula and the ACC have been observed (Steward and Chib 2024).

According to the results of our study, the two parameters that showed no difference between 60 and 100 trials were accuracy and velocity. Dysfunctions in the basal ganglia, parietal cortex, premotor neural networks, and specific cerebellar systems (Jensen et al. 2019), along with impairments in inhibitory mechanisms, can lead to incorrect target identification and saccadic accuracy (Munoz et al. 2003; Derakshan et al. 2009). Saccadic velocity is associated with the SC, mesencephalic reticular formation, pontine paramedian reticular formation, the condition of extraocular muscles, and motor neurons. It is primarily determined by premotor circuits in the brainstem and is less influenced by higher order cognitive control processes (Scudder et al. 2002). Given this, velocity and accuracy rely less on inhibitory mechanisms compared to latency and directional errors and are primarily governed by motor control. This finding may be attributed to the inhibitory effect having a lesser impact on accuracy and velocity than on latency and directional errors.

To the best of the authors' knowledge, no specific research has examined the effects of prolonged task duration on antisaccade performance. However, some studies have explored the relationship between response inhibition and mental fatigue. Guo et al. (2018) designed a simulated driving task to induce mental fatigue and investigated its negative effects on response inhibition in a visual Go/NoGo task. While the Go/NoGo task does not fully overlap with the antisaccade mechanism, its findings were examined due to the involvement of shared neural connections, including the right inferior frontal cortex, DLPFC, and ACC. In this study, an increase in reaction time and error rate was observed following the fatigue manipulation. The researchers suggested that these findings indicate that mental fatigue may slow down the inhibitory process (Guo et al. 2018). In another study, mental fatigue was induced by subjecting participants to a demanding 20‐min psychomotor vigilance test. In the antisaccade task administered afterward, participants in the fatigue condition exhibited significantly longer latency times compared to their pre‐fatigue state (Ahn et al. 2023). Additionally, it has been suggested that fatigue‐related performance decline may be linked to a reduced motivation to exert cognitive effort (Wójcik and Maj 2025).

### 4.2 Effects of Varying Trial Numbers on Prosaccade Task Performance

When examining the study results in terms of prosaccade outcome measures, no changes were observed with an increase in the number of trials. Since prosaccade generation is an automatic process primarily controlled by SC, FEF, and brainstem premotor circuits, it is less influenced by changes in inhibitory control (Munoz and Everling 2004). Prosaccades are generally rapid and accurate as they rely on a direct sensorimotor transformation mechanism. Given that the prosaccade task necessitates the involvement of relatively few brain regions to visually track a moving target (Hallett 1978), these findings suggest that the performance is not significantly impacted by the increase in trial numbers, likely due to the minimal role of inhibitory control in prosaccades.

## 5 Conclusion

A wide range of individuals who require an assessment of their cognitive functions may potentially benefit from the antisaccade test. There is an ongoing need to develop accurate procedures for administering the antisaccade test. In our study, increasing the number of trials and, consequently, the test duration led to a decline in antisaccade performance. Based on the demonstrated links between brain regions involved in executive functions that contribute to response inhibition in antisaccade responses and mental fatigue, we propose that an increased number of trials may impair antisaccade performance due to its association with mental fatigue. Among the antisaccade parameters, the greater impairment observed in outcome measures more closely related to executive functions, such as latency and error rate, compared to other measures such as velocity, may suggest an impact on the inhibition mechanism. Although the data from this study provide evidence that variations in the number of trials in the antisaccade test can influence test outcomes, further evidence is needed to fully understand the relationship between mental fatigue and the number of antisaccade trials. Additionally, potential impairments in the structures involved in antisaccade generation may result in an inability to meet the task demands, leading to a greater impact of fatigue. Following the data obtained from young and healthy individuals, applying the same method to pathological groups could be useful for evaluating its potential in early and differential diagnosis of various pathologies.

### 5.1 Limitations

There are some limitations to consider in this study. First, the 18–40 age range was initially treated as a relatively homogeneous group. However, age‐related effects may still influence performance to some extent. Dividing participants into 10‐year age groups could improve the precision of subgroup analyses. In future research, statistically controlling for age or conducting more detailed age‐based analyses may help enhance the validity and interpretability of the findings. Second, participants were assessed using single‐task conditions. To better understand the relationship between mental fatigue and antisaccade performance, including different cognitive load conditions in the test may provide a more comprehensive understanding. Finally, to ensure similar fatigue conditions, each participant first completed 100 trials of the prosaccade‐antisaccade task, followed by 60 trials of the prosaccade‐antisaccade task. Evaluating the effect of randomizing the test assignments may be useful to determine whether it makes any difference in performance outcomes.

## Author Contributions

**Aysenur Kucuk Ceyhan**: conceptualization, investigation, writing – original draft, methodology, validation, writing – review and editing, resources, data curation, visualization, project administration, formal analysis. **Asya Fatma Men**: methodology, investigation, writing – review and editing, data curation, validation. **Zahra Polat**: methodology, validation, writing – review and editing.

## Conflicts of Interest

The authors declare no conflicts of interest.

## Peer Review

The peer review history for this article is available at https://publons.com/publon/10.1002/brb3.70700

## Data Availability

Data may be available from the corresponding author upon reasonable request.
